# Clinical Profile and Reported Functional Impact of Primary Headache Disorders in Children Attending a Tertiary Care Hospital in South India

**DOI:** 10.7759/cureus.117759

**Published:** 2026-10-11

**Authors:** Shreeja Y, RV Jeya Balaji, Mohamed Azarudeen J, Karthik Raj

**Affiliations:** 1 Pediatrics, Upgraded Government Primary Health Centre, Chinnatharapuram, IND; 2 Pediatrics, Velammal Medical College Hospital and Research Institute, Madurai, IND

**Keywords:** adolescents, children, migraine, pain severity, pediatric neurology, primary headache, school absenteeism, screen time, tension-type headache

## Abstract

Background: Primary headache disorders are among the most common neurological conditions affecting children and adolescents and are associated with recurrent pain, school-related difficulties, and reduced quality of life. Although migraine and tension-type headache (TTH) are the predominant pediatric headache phenotypes, data from hospital-based settings in South India remain limited. This study aimed to determine the distribution of primary headache phenotypes and describe their clinical characteristics, associated symptoms, pain severity, lifestyle-related factors, and reported school-related indicators among children attending a tertiary-care hospital.

Methods: A hospital-based cross-sectional analytical study was conducted in the Department of Paediatrics, Velammal Medical College Hospital and Research Institute, Madurai, Tamil Nadu, India. A total of 220 children aged 6-15 years with primary headache disorders were enrolled using consecutive sampling. Demographic characteristics, headache profile, associated symptoms, screen exposure, precipitating and relieving factors, school-related indicators, nutritional status, and pain severity were assessed using a structured questionnaire and clinical evaluation.

Results: Among the 220 participants, the median age was 14 years, and 125 (56.8%) were female. Tension-type headache was the most common phenotype, occurring in 110 (50.0%) children, followed by migraine in 65 (29.5%), chronic persistent headache in 24 (10.9%), and other primary headache disorders in 21 (9.5%). Most children, 163 (74.1%), experienced headaches on 1-15 days per month, and the mean Visual Analog Scale (VAS) pain score was 5.28 ± 1.97. Moderate and severe pain were reported by 122 (55.5%) and 52 (23.6%) participants, respectively, while 113 (51.4%) children reported medication use for headache relief. Visual disturbances were reported in 49 (22.3%) children, followed by sensitivity to sound and nausea/vomiting as other commonly reported associated symptoms. Reported scholastic difficulties were observed in 119 (54.1%) children, and school absenteeism was frequently reported.

Conclusion: Tension-type headache was the predominant primary headache phenotype in this tertiary-care pediatric cohort. Primary headache disorders were associated with considerable clinical burden, including moderate-to-severe pain, medication use, reported scholastic difficulties, and school absenteeism. Screen exposure was a commonly reported lifestyle characteristic requiring consideration during clinical assessment rather than an established determinant of headache severity. Comprehensive management strategies incorporating accurate symptom recognition, lifestyle counseling, sleep optimization, nutritional assessment, stress management, and evaluation of reported school-related indicators may support appropriate headache care among affected children.

## Introduction

Primary headache disorders are common neurological conditions in children and adolescents and represent an important source of recurrent pain and functional impairment. A recent systematic review and meta-analysis estimated the pooled prevalence of overall primary headache disorders among children and adolescents at approximately 62%, with tension-type headache and migraine accounting for substantial proportions of cases [1]. Beyond pain itself, recurrent headaches can adversely affect physical functioning, psychological well-being, social participation, school attendance, and overall quality of life [2,3]. The resulting burden may extend to families through repeated healthcare visits, disruption of daily activities, and the need for ongoing symptom management.

Migraine and tension-type headache are the predominant primary headache phenotypes in the pediatric population, although their clinical presentation may vary according to age and developmental stage [1,3]. Younger children may have difficulty describing the location, character, duration, and associated features of headache, making a structured clinical history particularly important. Symptoms such as nausea, vomiting, photophobia, phonophobia, visual disturbances, and activity limitation can assist in distinguishing migraine from tension-type headache and other primary headache disorders. Standardized diagnostic classification is therefore essential for accurate phenotyping, appropriate management, and the exclusion of secondary causes of headache [4].

The burden of pediatric headache is influenced not only by headache phenotype and pain intensity but also by potentially modifiable behavioral and environmental factors. Sleep disruption, academic stress, irregular meals, reduced physical activity, and prolonged exposure to digital screens have been examined as possible correlates of recurrent headache [1-5]. A scoping review of 48 studies found that longer screen-use duration was the screen-related factor most consistently associated with pediatric headache; however, the available evidence was predominantly cross-sectional and showed substantial variation in device type, exposure measurement, and headache outcomes [5]. Consequently, screen exposure should be considered a potential correlate of headache burden rather than an established causal factor.

Indian studies have demonstrated a considerable burden of primary headache among school-aged children. A school-based study from Kashmir reported an overall prevalence of 66.4%, with tension-type headache and migraine as the principal phenotypes, and observed an increase in headache occurrence with age and a female predominance [3,6]. Clinical data from South India have also described primary headache and its associated factors among children attending hospital services [2,3,6,7]. Nevertheless, detailed tertiary-care data jointly examining headache phenotype, associated symptoms, pain severity, screen exposure, medication requirement, activity limitation, scholastic performance, and school absenteeism remain comparatively limited.

Therefore, the present study aimed to determine the distribution and clinical characteristics of primary headache disorders among children aged 6-15 years attending a tertiary care hospital in Madurai, South India. 

## Materials and methods

Study design and setting

This hospital-based cross-sectional analytical study was conducted in the Department of Pediatrics, Velammal Medical College Hospital and Research Institute, Madurai, Tamil Nadu, India. The study was carried out from September 2023 to August 2025 among children presenting with headache to the pediatric outpatient department. The primary objective was to determine the distribution of primary headache phenotypes among children aged 6-15 years. Secondary objectives included evaluation of clinical characteristics, associated symptoms, lifestyle-related factors, reported functional indicators, and exploratory associations between headache phenotype and selected clinical and lifestyle variables.

Study population

Children aged 6-15 years presenting with headache to the pediatric outpatient department during the study period were screened for eligibility. Participants fulfilling the diagnostic criteria for primary headache disorders were enrolled consecutively after obtaining informed consent from parents or legal guardians and assent from children when appropriate.

Inclusion and exclusion criteria 

Children aged 6-15 years who presented with recurrent headache and fulfilled the diagnostic criteria for primary headache disorders were eligible for inclusion. Written informed consent was obtained from parents or legal guardians, and child assent was obtained wherever applicable. Children were excluded if headache was attributable to secondary causes such as infection, trauma, intracranial lesions, metabolic disorders, systemic illness, or other identifiable medical conditions. Children with known neurological disorders, including epilepsy, neurodevelopmental disorders, or established intracranial pathology, were also excluded. Those requiring emergency management for acute medical conditions and those with incomplete clinical records or missing essential study variables were not included in the final analysis.

Clinical evaluation and exclusion of secondary headache

All children were evaluated by structured clinical history followed by general physical and neurological examination before classification of the headache disorder. The clinical assessment sought features suggestive of secondary headache, including acute systemic illness or infection, recent head trauma, known neurological disease, established intracranial pathology, medication- or substance-related headache, and other identifiable systemic or metabolic conditions. Children with a clinically identifiable secondary cause, abnormal findings requiring further evaluation, or an acute condition requiring emergency management were excluded from the primary-headache cohort. Headache disorders were subsequently classified according to the International Classification of Headache Disorders, Third Edition (ICHD-3)-based clinical assessment.

Neuroimaging and laboratory investigations were not mandated routinely for all participants as part of the study protocol. Additional diagnostic investigations were undertaken as clinically indicated during routine patient care when the history or examination raised suspicion of a secondary cause.

Sample size and sampling technique

The sample size was estimated using the formula for a single population proportion. Based on a previous Indian study reporting a proportion of primary headache disorders of 66.4%, with a 95% confidence level and an absolute precision of 6.5%, the minimum required sample size was 203 participants. The target sample was increased to 220 to account for potentially incomplete observations [4]:

$n=\frac{Z_{1-\alpha/2}^{2}\times p(1-p)}{d^{2}}$

where n represents the required sample size.

$n=\frac{(1.96)^{2}\times0.664\times(1-0.664)}{(0.065)^{2}}=202.86\approx203$

Analyses examining associations between headache phenotype and clinical variables, and between lifestyle factors such as screen exposure and pain severity, were secondary exploratory analyses and were not used in the original sample-size calculation. With N = 220, a chi-square analysis of a 3 × 4 contingency table has approximately 80% power at α = 0.05 to detect an overall effect of approximately Cramer's V = 0.25; therefore, the study was more capable of detecting moderate than small associations. Analyses involving less frequent headache phenotypes should be interpreted cautiously because of the smaller subgroup sizes. Eligible children aged 6-15 years presenting with recurrent headache and fulfilling the inclusion criteria were recruited consecutively until the required sample size was achieved.

Diagnostic classification

Headache phenotypes were assigned using the ICHD-3 as the diagnostic framework. The structured research proforma recorded headache site and laterality, character, duration, monthly frequency, pain severity, timing, associated symptoms, precipitating and relieving factors, and reported functional impact. Final phenotype assignment was made by the evaluating pediatric clinician after integrating the structured history and clinical examination rather than by an automated algorithm based solely on individual proforma fields [8,9].

Migraine classification was based on a compatible recurrent headache pattern with appropriate duration, pain characteristics, and severity, together with typical associated features such as nausea/vomiting and/or photophobia and phonophobia; aura was recorded separately when present. Tension-type headache was assigned when the headache pattern was compatible with the ICHD-3 tension-type phenotype, including characteristic duration, pressing/tightening quality, appropriate severity, and absence of features sufficient for another primary headache diagnosis. Chronic persistent headache and other primary headache phenotypes were classified based on the clinician's assessment and available headache characteristics. Secondary causes were excluded clinically as described above [5]. Because the study proforma was not a verbatim ICHD-3 criterion checklist, not every diagnostic criterion was retained as an independently analyzable study variable. Therefore, phenotype classification represents the contemporaneous clinician-assigned diagnosis based on ICHD-3-informed clinical evaluation rather than retrospective fulfillment of every individual diagnostic criterion.

Data collection

Data were collected using an investigator-developed structured questionnaire administered through direct interviews with the child and parent/guardian, followed by a detailed clinical evaluation (see Appendices). Information on demographic characteristics, including age, sex, place of residence, type of school attended, and socioeconomic status, was recorded. Clinical headache characteristics were documented, including age at onset, frequency and duration of headache episodes, site and nature of pain, timing of headache occurrence, aggravating and relieving factors, and medication use. Associated symptoms such as nausea, vomiting, photophobia, phonophobia, visual disturbances or aura, rhinorrhea, and other accompanying manifestations were assessed. Information regarding reported lifestyle and environmental factors, including daily screen-time exposure, sleep patterns, perceived academic stress, physical activity, dietary habits, and other reported precipitating factors, was also collected.

To evaluate the reported functional impact of headache, data regarding school absenteeism, reported scholastic difficulties, headache-related medication use, and relevant lifestyle factors were obtained. Anthropometric measurements, including height and weight, were recorded using standard procedures, and nutritional status was assessed according to age-appropriate growth standards.

Medication use was recorded as a clinical history variable based on caregiver/child reports of whether medication had been used for headache relief. Detailed information regarding medication class, dosage, duration, and monthly frequency of medication use was not systematically collected. Therefore, medication-overuse headache was not assessed as a separate diagnostic outcome.

Scholastic performance was assessed through caregiver/child-reported history during clinical evaluation. It was recorded as reported academic difficulty or perceived scholastic difficulty and was not based on standardized examination scores, school records, or teacher assessments. Therefore, this variable represents a subjective indicator of reported functional impact rather than an objective measure of academic achievement.

School absenteeism was assessed based on the reported number of school days missed due to headache-related symptoms during a typical month. For analysis, absenteeism was categorized into mutually exclusive groups: 0-2 days/month, 3-7 days/month, 8-14 days/month, and >14 days/month.

Assessment of pain severity

Pain intensity was assessed using the Visual Analog Scale (VAS), ranging from 0 to 10 [6] (Table 6). Pain severity was categorized as: (1) mild (VAS score <4), (2) moderate (VAS score 4-6), and (3) severe (VAS score ≥7).

Anthropometric assessment

Height and weight were measured using standard techniques. Nutritional status was classified according to age-appropriate anthropometric standards as underweight, normal weight, or overweight.

Ethical considerations

The study protocol was approved by the Institutional Ethics Committee of Velammal Medical College Hospital and Research Institute, Madurai (IEC No. VMCIEC/041/2023). The study was conducted in accordance with the ethical principles outlined in the Declaration of Helsinki. Written informed consent was obtained from parents or legal guardians before enrollment. Assent was obtained from children whenever appropriate.

Statistical analysis

Data were entered into Microsoft Excel (Microsoft Corporation, Redmond, Washington) and analyzed using Jamovi Statistics Version 2.6.19 (The jamovi Project, Sydney, Australia). Continuous variables were tested for normality and summarized as mean ± standard deviation (SD) or median with interquartile range (IQR), as appropriate. Categorical variables were presented as frequencies and percentages. Associations between headache phenotype and categorical variables were assessed using the chi-square test or Fisher's exact test where applicable. Comparisons of continuous variables across headache groups were performed using one-way analysis of variance (ANOVA) or the Kruskal-Wallis test depending on data distribution. The association between screen-time exposure and pain severity was evaluated using both categorical comparison (chi-square test between screen-time categories and pain severity categories) and correlation analysis using Spearman's rank correlation coefficient for continuous screen-time and VAS scores. A two-tailed p-value <0.05 was considered statistically significant.

## Results

Baseline demographic profile

A total of 220 children aged 6-15 years who fulfilled the clinical criteria for primary headache disorders were included in the analysis. The median age of participants was 14 years. Females constituted 125 (56.8%) participants, while males accounted for 95 (43.2%). Most participants belonged to the middle socioeconomic category (147, 66.8%). Government-school students represented 177 (80.5%) participants, and 162 (73.6%) children were from urban areas. Anthropometric assessment showed that 132 (60.0%) participants were classified as underweight, 66 (30.0%) had normal nutritional status, and 22 (10.0%) were overweight (Table 1). 

**Table 1:** Baseline demographic and anthropometric characteristics of the study participants (N = 220).

Characteristics	Category	n (%) or value
Age	Median	14 years
Gender	Female	125 (56.8%)
	Male	95 (43.2%)
School type	Government school	177 (80.5%)
	CBSE	33 (15.0%)
	International school	10 (4.5%)
Residence	Urban	162 (73.6%)
	Rural	58 (26.4%)
Socioeconomic status	Middle class	147 (66.8%)
Nutritional status	Underweight	132 (60.0%)
	Normal	66 (30.0%)
	Overweight	22 (10.0%)

Distribution of primary headache phenotypes

Tension-type headache was the most common primary headache phenotype, observed in 110 children (50.0%). Migraine was identified in 65 children (29.5%). Chronic persistent headache accounted for 24 (10.9%) participants, while other primary headache disorders accounted for 21 (9.5%). Among children with migraine, 57 (88.0%) had migraine without aura, and 8 (12.0%) had migraine with aura (Table 2).

**Table 2:** Distribution of primary headache phenotypes among study participants.

Headache phenotype	n (%)
Tension-type headache	110 (50.0%)
Migraine	65 (29.5%)
Chronic persistent headache	24 (10.9%)
Other primary headache disorders	21 (9.5%)
Total	220 (100.0%)
Migraine subtype	
Migraine without aura	57 (88.0%)
Migraine with aura	8 (12.0%)
Total migraine cases	65 (100.0%)

Clinical headache profile

Most children reported headaches occurring on 1-15 days per month, with 163 participants (74.1%) falling within this frequency category. Headaches occurred most commonly in the evening, as reported by 115 children (52.3%). The most frequently reported headache sites were the back of the head in 57 children (25.9%) and the temples in 53 children (24.1%).

Tight or pressing pain was the predominant headache character, reported by approximately two-thirds of participants. The mean VAS pain score was 5.28 ± 1.97. Based on predefined VAS categories, moderate pain was reported by 122 (55.5%) participants, while severe pain was reported by 52 (23.6%). A total of 113 (51.4%) children reported using medication for headache relief (Table 3).

**Table 3:** Clinical headache characteristics, pain severity, medication use, and reported school-related indicators.

Variable	Category/Statistic	n (%) or value
Headache frequency	1-15 days/month	163 (74.1%)
Time of occurrence	Evening	115 (52.3%)
Common headache site	Back of head	57 (25.9%)
Common headache site	Temples	53 (24.1%)
VAS pain score	Mean ± SD	5.28 ± 1.97
Pain severity	Mild	46 (20.9%)
Pain severity	Moderate	122 (55.5%)
Pain severity	Severe	52 (23.6%)
Medication use for headache relief	Yes	113 (51.4%)
Reported scholastic difficulties	Yes	119 (54.1%)
School days missed due to headache	0-2 days/month	19 (8.6%)
	3-7 days/month	41 (18.6%)
	8-14 days/month	91 (41.4%)
	>14 days/month	69 (31.4%)

Associated symptoms

Visual disturbances were the most commonly reported associated symptom, occurring in 49 children (22.3%), followed by sensitivity to sound and nausea/vomiting. Aura was reported exclusively among children classified with migraine. Nausea/vomiting was frequently reported among children with migraine and tension-type headache, while sensitivity to light, sensitivity to sound, and visual disturbances were reported across different headache phenotypes. The distribution of associated symptoms differed significantly across headache phenotypes (χ² = 10.3, df = 10, p = 0.001; Cramer's V = 0.153) (Table 4).

**Table 4:** Summary of statistical associations.

Association tested	Result
Associated symptoms vs headache phenotype	p = 0.001
Pain severity vs headache phenotype	p = 0.045
Screen time vs pain severity	χ² = 0.440, p = 0.979; Spearman ρ = −0.015, p = 0.823
Screen time vs headache phenotype	p = 0.257
School absenteeism vs headache phenotype	p = 0.173
Family income vs headache phenotype	p = 0.643
Hobbies vs headache phenotype	p = 0.975
Relieving factors vs headache phenotype	p = 0.334
Precipitating factors vs headache phenotype	p = 0.833

Lifestyle factors, triggers, and relieving factors

Screen exposure was a commonly reported lifestyle characteristic in this cohort, with the largest proportion of children reporting 2-4 hours of daily screen time. Stress and academic burden were among the most frequently reported precipitating factors. Sleep was the most commonly reported relieving factor, reported by 97 children (44.1%), followed by resting in a quiet place, reported by 86 children (39.1%).

Reported school-related indicators and anthropometry

School-related indicators were frequently reported in this cohort. Scholastic difficulties were reported in 119 (54.1%) children. This variable was based on caregiver/child-reported information and was interpreted as an indicator of perceived school-related impact rather than an objective measure of academic performance. School absenteeism was also commonly reported, with 19 (8.6%) children missing 0-2 school days/month, 41 (18.6%) missing 3-7 days/month, 91 (41.4%) missing 8-14 days/month, and 69 (31.4%) missing more than 14 days/month (Table 3). Underweight status was present in 132 children (60.0%).

Association analyses

Pain severity categories showed a statistically significant association with headache phenotype (p = 0.045), indicating differences in the distribution of mild, moderate, and severe pain categories across headache groups. The distribution of associated symptoms also differed significantly across headache phenotypes (χ² = 10.3, df = 10, p = 0.001; Cramer's V = 0.153). Aura was observed only among children classified as having migraine, while other associated symptoms were reported across different headache phenotypes.

Screen exposure was not significantly associated with pain severity. Categorical comparison of screen-time groups with pain severity demonstrated no significant association (χ² = 0.440, p = 0.979; Cramer's V = 0.0316). Similarly, Spearman correlation analysis showed no significant correlation between screen-time score and VAS pain score (ρ = −0.015, p = 0.823). Screen exposure was also not significantly associated with headache phenotype (p = 0.257). Other evaluated variables, including school absenteeism (p = 0.173), family income (p = 0.643), hobbies (p = 0.975), relieving factors (p = 0.334), and precipitating factors (p = 0.833), did not demonstrate statistically significant associations with headache phenotype (Table 4).

## Discussion

This hospital-based cross-sectional study evaluated the distribution, clinical characteristics, pain intensity, associated symptoms, lifestyle-related factors, and reported functional indicators of primary headache disorders among 220 children aged 6-15 years presenting to a tertiary-care pediatric outpatient department in Madurai, South India. Tension-type headache was the most common phenotype, followed by migraine, chronic persistent headache, and other primary headache disorders. The cohort demonstrated considerable clinical burden, including moderate-to-severe pain, frequent medication use, reported school-related difficulties, frequent screen exposure, and a high proportion of underweight children. Significant associations were observed between headache phenotype and pain severity and between headache phenotype and associated symptoms. However, screen exposure was not significantly associated with headache phenotype or pain severity. These findings highlight that pediatric primary headache requires structured clinical evaluation incorporating symptom characterization, lifestyle assessment, and evaluation of reported school-related impact.

The predominance of tension-type headache in the present cohort is consistent with existing evidence identifying tension-type headache and migraine as the major primary headache phenotypes in children and adolescents [1-3]. In this study, tension-type headache accounted for half of the cases, while migraine accounted for nearly one-third. However, this distribution reflects the pattern of headache phenotypes among healthcare-seeking children rather than prevalence in the general pediatric population. Community- and school-based studies evaluate headache occurrence among broader pediatric populations, whereas hospital-based cohorts include children whose symptoms were sufficiently concerning to require clinical assessment [2,3]. Therefore, these findings should not be generalized directly to all children in the community.

In the current study, most participants were older children or adolescents, with a slight female predominance. Previous epidemiological studies have demonstrated that headache frequency increases with age and that sex differences become more evident during adolescence [1,3]. Pubertal development, hormonal changes, academic demands, psychosocial stress, sleep disruption, and changing electronic-device use patterns may represent relevant lifestyle characteristics associated with pediatric headache assessment [8,10,11]. However, because this was a hospital-based cohort, the observed female predominance should not be interpreted as an independent population-level risk factor.

Pain severity represented an important clinical characteristic in this cohort. The mean VAS score indicated moderate overall pain intensity, and a substantial proportion of children reported medication use for symptom relief. Although variations in pain severity were observed across headache phenotypes, pain intensity alone cannot reliably distinguish headache disorders because pain reporting may be influenced by age, developmental stage, psychological factors, parental perception, and recall. Therefore, pain assessment should complement, rather than replace, standardized clinical phenotyping and diagnostic evaluation [8,9].

Associated symptoms such as nausea, vomiting, photophobia, phonophobia, visual symptoms, and autonomic complaints are clinically relevant features in pediatric headache assessment. Migraine commonly includes gastrointestinal and sensory symptoms, while autonomic manifestations may occur in selected headache phenotypes [8,9]. In the present study, these symptoms were documented as part of clinical characterization; however, differences between headache phenotypes should be interpreted cautiously because the study proforma was designed to capture major clinical features rather than a complete criterion-by-criterion diagnostic checklist for every headache subtype. Structured questioning regarding associated symptoms remains important because younger children may not spontaneously report these features [8,9].

Accurate classification of uncommon headache phenotypes requires particular attention. On re-evaluation of phenotype classification, the subgroup previously labeled as cluster headache (24/220; 10.9%) was identified as chronic persistent headache rather than cluster headache. Therefore, cluster headache-specific interpretation has been removed from the revised manuscript. This highlights the importance of accurate phenotype classification and comprehensive diagnostic documentation in pediatric headache research. Future studies evaluating less common headache phenotypes should incorporate dedicated ICHD-3 diagnostic checklists, detailed phenotype-specific variables, and specialist headache assessment where appropriate.

Screen exposure was a commonly observed lifestyle characteristic in this cohort. A substantial proportion of participants reported daily electronic-device use, and previous studies have suggested that screen-related behaviors may be associated with pediatric headache, although findings remain heterogeneous because studies differ in device type, duration of exposure, posture, sleep effects, and headache outcomes [10]. Population-based research has also identified screen use and other lifestyle behaviors as relevant correlates of recurrent headache among children and adolescents [11]. In the present study, screen exposure should be interpreted as a potentially relevant behavioral characteristic rather than an established determinant of headache severity or phenotype.

The analysis did not demonstrate a significant association between screen exposure and headache phenotype or pain severity. Furthermore, the cross-sectional design prevents assessment of temporal relationships. Increased screen exposure may influence headache-related symptoms through mechanisms such as sleep disruption, reduced physical activity, or prolonged visual and postural strain; conversely, children experiencing recurrent headaches may modify their activities and spend more time using electronic devices during periods of reduced activity [10,11]. Therefore, screen exposure should be considered within the broader clinical and behavioral context rather than interpreted as a direct cause of headache-related symptoms.

The study demonstrated considerable reported school-related impact among children with primary headache. More than half of the participants had reported scholastic difficulties, and school absenteeism was frequently reported. Previous studies have demonstrated associations between primary headache disorders and impaired school attendance, reduced participation in activities, and diminished quality of life among children and adolescents [12,13]. However, scholastic difficulties and absenteeism in the present study were based on reported information rather than independently verified school records. Academic outcomes may also be influenced by socioeconomic circumstances, sleep patterns, nutritional status, family stress, school pressure, and emotional or behavioral factors.

The high proportion of underweight children was another notable observation. However, the absence of a headache-free comparison group prevents determination of whether underweight status is specifically associated with primary headache. Additionally, detailed dietary intake, hydration status, biochemical nutritional markers, and longitudinal nutritional changes were not assessed. Therefore, the nutritional findings should be considered descriptive, and no causal relationship between undernutrition and headache can be inferred. Future studies should incorporate standardized nutritional assessment and detailed dietary evaluation [14].

Stress, academic issues, and lifestyle-related factors were commonly reported precipitating factors, while sleep and rest in a quiet environment were frequently reported relieving factors. These findings are consistent with the biopsychosocial understanding of pediatric headache and support the importance of addressing sleep hygiene, hydration, meal regularity, physical activity, stress management, and individualized trigger identification during clinical care [8,11]. Sleep assessment is particularly relevant because sleep disturbance may function both as a precipitating factor and a consequence of recurrent headache.

The clinical implications of this study are practical. Children presenting with recurrent headache should undergo structured evaluation using standardized diagnostic criteria [8,9]. Detailed assessment of headache characteristics, associated symptoms, pain severity, and reported functional indicators can assist clinical classification and management planning. Counseling may include discussion of screen-use practices, regular sleep schedules, hydration, meal regularity, physical activity, stress management, and attention to school functioning as components of holistic headache care [8,10,11]. Children with neurological warning signs, progressive worsening of symptoms, frequent disabling attacks, significant reported functional difficulties, or poor response to initial management should be considered for specialist evaluation [8].

A validated pediatric headache disability instrument such as PedMIDAS was not administered in this study; therefore, standardized headache-related disability could not be quantified. The reported functional indicators assessed in this study, including school absenteeism, reported scholastic difficulties, medication use, and pain intensity, should therefore be interpreted as clinical and school-related indicators rather than validated disability measures. Furthermore, multivariable analyses were not performed because the study was primarily designed for descriptive characterization and exploratory association analysis. Therefore, potential confounding factors such as sleep duration, psychological stress, pubertal status, socioeconomic factors, and physical activity could not be independently evaluated. Future studies incorporating validated disability instruments, objective functional assessments, larger sample sizes, and multivariable analytical approaches are required to identify independent predictors of pediatric headache outcomes.

## Conclusions

Tension-type headache was the most common primary headache phenotype among children aged 6-15 years attending this tertiary-care pediatric outpatient clinic, followed by migraine and other primary headache disorders. Primary headache disorders were associated with considerable clinical burden and reported school-related impact, including moderate-to-severe pain, medication use, school absenteeism, and reported scholastic difficulties. The findings emphasize the importance of structured clinical assessment of pediatric headache, including accurate phenotype classification, evaluation of associated symptoms, assessment of pain severity, and identification of relevant lifestyle and school-related indicators. Comprehensive management approaches incorporating headache education, evaluation of lifestyle factors, sleep optimization, nutritional assessment, stress management, and individualized counseling may support appropriate clinical care and improve outcomes among children with primary headache disorders.

## Figures and Tables

**Figure 1:**
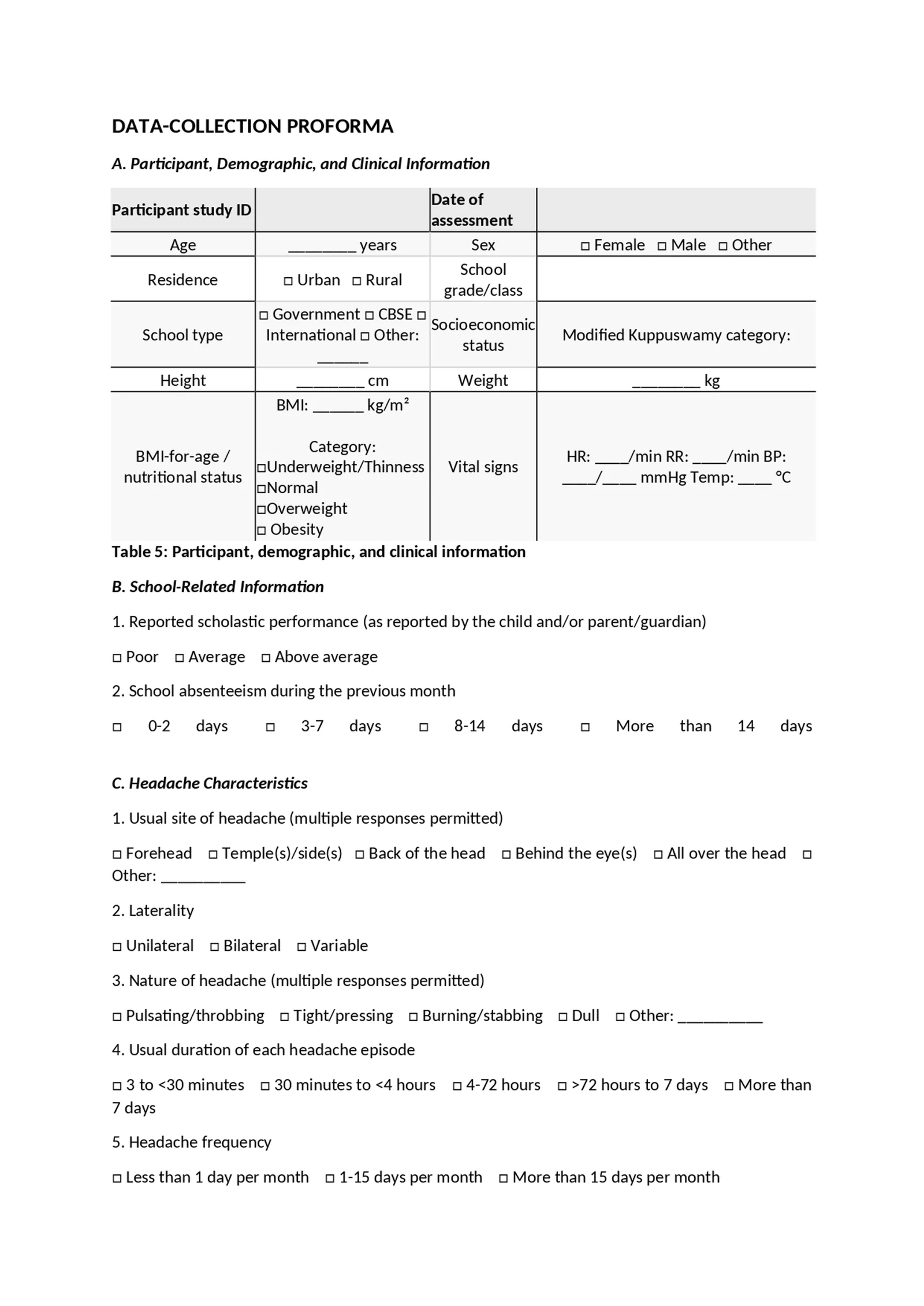
Data-collection proforma (Part 1)

**Figure 2:**
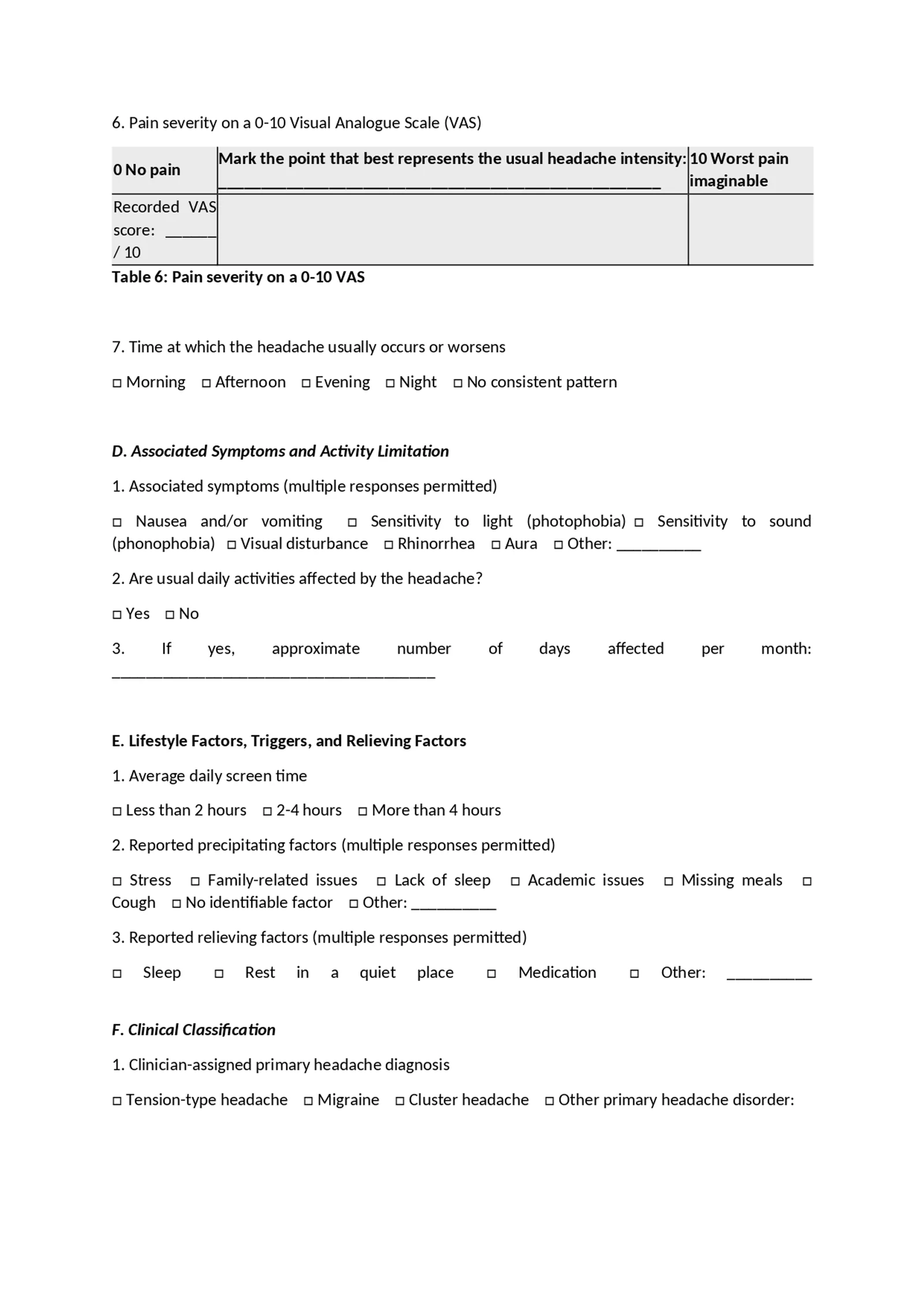
Data-collection proforma (Part 2)

## References

[1] Onofri A, Pensato U, Rosignoli C (2023). Primary headache epidemiology in children and adolescents: a systematic review and meta-analysis. J Headache Pain.

[2] Hassan AR, Palani A, James S, Manokaran RK, Arunprasath TS (2022). Primary headache in children aged between 6 and 18 years-causes and associated factors. Indian J Pediatr.

[3] Malik AH, Shah PA, Yaseen Y (2012). Prevalence of primary headache disorders in school-going children in Kashmir Valley (north-west India). Ann Indian Acad Neurol.

[4] Naing L, Nordin RB, Abdul Rahman H, Naing YT (2022). Sample size calculation for prevalence studies using Scalex and ScalaR calculators. BMC Med Res Methodol.

[5] Olesen J (2018). International classification of headache disorders. Lancet Neurol.

[6] Delgado DA, Lambert BS, Boutris N, McCulloch PC, Robbins AB, Moreno MR, Harris JD (2018). Validation of digital visual analog scale pain scoring with a traditional paper-based visual analog scale in adults. J Am Acad Orthop Surg Glob Res Rev.

[7] Hershey AD, Powers SW, Vockell AL, LeCates S, Kabbouche MA, Maynard MK (2001). PedMIDAS: development of a questionnaire to assess disability of migraines in children. Neurology.

[8] Orr SL (2024). Headache in children and adolescents. Continuum (Minneap Minn).

[9] (2018). Headache Classification Committee of the International Headache Society (IHS) The International Classification of Headache Disorders, 3rd edition. Cephalalgia.

[10] Langdon RL, DiSabella MT, Strelzik JA (2024). Screen time and pediatric headache: a scoping review of the literature. Headache.

[11] Nilles C, Williams JV, Patten SB, Pringsheim TM, Orr SL (2024). Lifestyle factors associated with frequent recurrent headaches in children and adolescents: a Canadian population-based study. Neurology.

[12] Al-Hashel JY, Alroughani R, Shuaibi S (2020). Impact of primary headache disorder on quality of life among school students in Kuwait. J Headache Pain.

[13] Magnerou AM, Doumbe JN, Paule Rose DT (2024). Prevalence and impact of primary headaches among students aged 8-12 years in Sub-Saharan Africa: Cameroon experience. Cephalalgia.

[14] Magdy R, Hassan A, Mohammed Z, Abdeltwab MA, Ghaffar NF, Hussein M (2024). Validity and reliability of Arabic version of pediatric migraine disability assessment scale (Child Self-Report versus Parent Proxy-Report): a multi-center study. J Headache Pain.

